# Influenza Vaccination Appropriateness: Insights from the Local Health Unit of Catania During the 2023/2024 and 2024/2025 Seasons

**DOI:** 10.3390/vaccines13090925

**Published:** 2025-08-30

**Authors:** Francesco Leonforte, Claudio Fiorilla, Gabriele Giorgianni, Vito Nicosia, Fabio Contarino, Cristina Genovese, Giovanni Genovese, Giustino Morlino, Martina Chimienti, Antonio Mistretta

**Affiliations:** 1 Department of Integrated Hygiene, Organizational, and Service Activities (Structural Department), Health Management, University Hospital Polyclinic “G. Rodolico—San Marco”, 95123 Catania, Italy; 2 Department of Public Health, Syracuse Local Health Authority, 96100 Siracusa, Italy; claudio.fiorilla@gmail.com (C.F.); fabiocontarino@hotmail.it (F.C.); 3 Public Health Unit, Epidemiology and Preventive Medicine (SEMP), Provincial Health Authority of Catania, 95124 Catania, Italy; gabriele.giorgianni@aspct.it; 4 Department of Medical and Surgical Sciences and Advanced Technologies “G. F. Ingrassia”, University of Catania, 95131 Catania, Italy; l98003432@studium.unict.it (V.N.); anmist@unict.it (A.M.); 5 Department of Biomedical, Dental and Morphological and Functional Imaging Sciences, University of Messina, 98122 Messina, Italy; crigenovese@unime.it; 6 Translational Molecular Medicine and Surgery, University of Messina, 98122 Messina, Italy; gigenovese@unime.it; 7 Accreditation and Supply Network Area, Local Health Authority “Roma 1”, 00193 Roma, Italy; gmiustus@gmail.com; 8 Department of Biomedicine and Prevention, University of Rome “Tor Vergata” and District and Commissioning Management, Local Health Authority “Roma 2”, 00133 Roma, Italy; martina.chimienti@gmail.com; 9 Scientific Communication Service, National Institute of Public Health (Istituto Superiore di Sanità), 00161 Roma, Italy

**Keywords:** influenza, vaccine, appropriateness, targeted risk, public health

## Abstract

**Background/Objectives**: Influenza poses a substantial global public health challenge, disproportionately affecting vulnerable populations. Vaccination is the most effective preventive measure, and recent strategies in Italy emphasize the principle of “appropriateness”—the alignment of specific vaccine formulations (e.g., adjuvanted or high-dose) with targeted risk groups to optimize protection. Nevertheless, challenges persist in ensuring the consistent administration of the most suitable vaccine, particularly among high-risk individuals who would benefit most. **Methods**: A retrospective descriptive study was conducted using data from the 2023–2024 and 2024–2025 influenza vaccination campaigns of the Local Health Authority of Catania. Vaccination data were analyzed by age group and vaccine type, based on national immunization guidelines. Population categories included individuals ≥ 65 years, adults 60–64 years, adults 18–59 years (with/without chronic conditions), children, and pregnant/postpartum women. Vaccine types analyzed were aQIV, QIV-HD, QIV-SD, QIVcc, and LAIV. Descriptive statistics were used, and Relative Risk (RR) with 95% Confidence Intervals (CI) was calculated using the 60–64 age group as a reference. Analyses were performed with Stata 18.0. **Results**: In 2023–2024, 78.8% of individuals ≥ 65 received recommended vaccines, compared to 100% in the 60–64 group (RR = 0.23; 95% CI: 0.225–0.231). Adults 18–59, children, and pregnant/postpartum women showed ≥99% adherence. In 2024–2025, appropriateness in the ≥65 group improved to 96.1% (RR = 0.12; 95% CI: 0.118–0.128). All other groups maintained high adherence (≥99%), except for 6.2% of children aged 6 months–2 years who inappropriately received LAIV. **Conclusions**: Despite dramatically improved vaccination appropriateness in the elderly, a persistent and critical safety issue--inappropriate administration LAIV use in 6.2% of young children—highlights the need for targeted interventions to ensure complete patient safety.

## 1. Introduction

Influenza is a highly contagious, acute viral respiratory infection that affects individuals of all ages worldwide, causing substantial morbidity and mortality, especially during seasonal epidemics and pandemics [1,2]. Each year, it infects approximately 10% of the global population and is responsible for up to 650,000 deaths, with the highest risk of severe disease observed in young children, older adults, and individuals with underlying health conditions [3,4,5]. People aged 65 years and older, as well as children under 5 years old, bear the greatest burden in terms of hospitalization and mortality [6]. Influenza leads to an estimated 290,000 to 650,000 respiratory-related deaths annually across the globe, most of which occur in older adults. In addition, it causes millions of hospital admissions and considerable economic costs due to healthcare expenditures and lost productivity [6,7]. The emergence of novel viral strains and resistance to antiviral medications further complicates treatment and prevention strategies [7,8].

Vaccination is widely acknowledged as the most effective tool for preventing influenza infection and its associated sequelae [9,10]. It significantly lowers the risk of severe illness, hospitalization, mortality, and life-threatening complications such as pneumonia, bacterial superinfections, acute respiratory distress syndrome (ARDS), and multi-organ failure, particularly among young children, older adults and patients with chronic diseases [11,12,13]. Moreover, vaccination lowers the need for intensive care in hospitalized patients [14,15].

In Italy, national influenza vaccination guidelines are revised annually to define priority groups and indicate the most appropriate vaccines according to age, health status, and expected efficacy. Recent recommendations have embraced the concept of “precision vaccination”, expanding eligibility criteria and customizing vaccine selection to enhance protection in high-risk groups [16]. Over time, these target populations have come to include children between 6 months and 6 years of age, adults aged 60 to 64, pregnant and postpartum women at any stage of pregnancy, adults over 65 years, and individuals with chronic diseases or elevated risk of complications [11,17,18].

Since the 2018–2019 influenza season, increasing attention has been placed on aligning each group with the most suitable vaccine. For older adults, in particular, adjuvanted and high-dose vaccines are recommended to enhance immunogenicity and clinical protection. This recommendation is rooted in the biological phenomenon of immunosenescence, the age-associated decline in immune system function that leaves older adults both more susceptible to severe influenza and less responsive to standard-dose vaccines. Enhanced formulations are therefore specifically designed to overcome this blunted immune response, thereby providing more robust protection [11,19]. In Italy, six main types of influenza vaccines are authorized and used during seasonal campaigns. These include QIV-SD, QIVcc, aQIV, QIV-HD; and LAIV, indicated for pediatric use [17,20,21].

In recent years, vaccination campaigns have increasingly focused on the concept of “appropriateness”—administering the most suitable vaccine for each individual based on age, risk profile, and clinical status, instead of applying a uniform approach [22,23]. This tailored strategy aims to improve immune response, particularly in individuals with reduced immunocompetence, contributes to better use of healthcare resources, and strengthens community-level protection by reducing severe cases and limiting transmission [11,22].

Immunization status, defined as receiving all recommended vaccines on time, is widely recognized as a key quality indicator in both pediatric and adult populations. High vaccination uptake rates reflect the effectiveness, efficiency, and equity of immunization programs [24]. However, gaps in coverage and reduced vaccine effectiveness persist in certain population groups, often due to suboptimal vaccine use, limited access to services, or specific clinical barriers [25].

To promote more precise and effective protection, the Italian Ministry of Health outlines the specific vaccine recommendations for each target group in its annual circulars. Nonetheless, challenges remain in ensuring the consistent delivery of the most appropriate vaccine formulations across all subgroups. In particular, suboptimal vaccines may still be administered to high-risk individuals, such as the elderly, who would benefit most from enhanced formulations like aQIV or QIV-HD [11,26].

In this context, the present study aims to assess the appropriateness of influenza vaccination within the population served by the Local Health Authority (LHA) of Catania. To the best of our knowledge, no population-level analyses have directly measured the appropriateness of influenza vaccine administration within a LHA. By aligning local data with national recommendations and recent European evidence, the study seeks to identify strengths and areas for improvement, ultimately contributing to the development of a more targeted and equitable vaccination program.

## 2. Materials and Methods

### 2.1. Study Design

This is a retrospective descriptive study based on data collected from the influenza vaccination campaign conducted by the LHA of Catania during the 2023–2024 and 2024–2025 seasons. Within the LHA, influenza vaccines are administered by general practitioners, pediatricians, and public health vaccination centers. All administrations are routinely recorded in the regional immunization registry. For the present analysis, aggregated data were extracted from this registry and organized according to the predefined age groups and population categories established by national immunization guidelines. The dataset did not include individual-level information, but only summary counts by group, which served as the basis for the descriptive and comparative analyses of vaccine administration by age group and vaccine type. For both seasons analyzed, the vaccine supply of the LHA of Catania included all types recommended by ministerial circulars (aQIV, QIV-HD, QIV-SD, QIVcc, and LAIV), alt-hough this analysis cannot rule out temporary local-level stock shortages that may have influenced provider choices.

### 2.2. Study Variables

The variables analyzed in this study included the population categories eligible for influenza vaccination and the type of vaccine recommended or suggested for each group. Population categories were defined in accordance with national immunization guidelines [12,27] and included individuals aged ≥65 years, adults aged 60–64 years, adults aged 18–59 years with or without chronic conditions, children of different age groups, and pregnant or postpartum women. In this study, a vaccine administration was defined as ‘inappropriate administration’ if the vaccine type used was not aligned with the explicit recommendations (indicated by ‘R’ in Table 1) or was contraindicated (‘-’) for that specific population group, according to national guidelines. The vaccine types considered were aQIV, QIV-HD, QIV-SD, QIVcc, and LAIV. Some professional subgroups (e.g., healthcare workers, essential service employees, caregivers of fragile patients) were included within the broader age- and risk-based categories, in line with Ministry of Health recommendations. A summary of vaccine recommendations by population category is provided in Table 1.

### 2.3. Statistical Analysis

Descriptive statistics were used to summarize vaccination data by population group and vaccine type. For each group, the median number of doses administered and standard deviation (SD) were calculated. For the calculation of Relative Risk (RR), the 60–64 years age group was chosen as the reference. This choice is motivated by the fact that, for this cohort, national guidelines permit the use of nearly all available vaccine formulations (except LAIV), making it easier to achieve 100% appropriateness. Therefore, this group represents an ideal benchmark to quantify deviations from the stricter recommendations applied to other groups, such as individuals aged ≥65 years. All analyses were performed using Stata, version 18.0 (StataCorp LP, College Station, TX, USA).

## 3. Results

### 3.1. 2023–2024 Campaign

**Figure 1:**
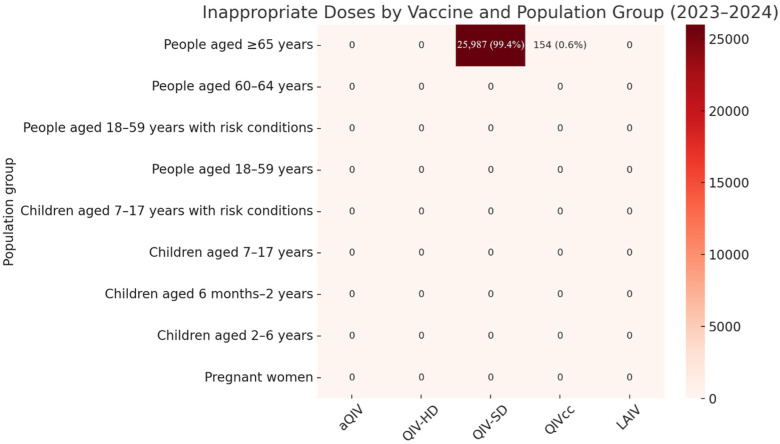
Distribution of inappropriate influenza vaccine administrations by population group and vaccine type during the 2023–2024 season. Note: each cell reports the number and percentage of non-recommended doses administered within the group. White cells indicate no inappropriate administrations.

A summary of the findings on inappropriate vaccine administrations is presented in Figure 1. During the 2023–2024 influenza season, a total of 123,281 individuals aged ≥65 years received vaccination. Among them, 21.2% were administered non-recommended formulations (25,987 QIV-SD and 154 QIVcc doses), instead of the recommended enhanced vaccines (aQIV or QIV-HD). Consequently, only 78.8% of vaccinations in this group were appropriate.

By contrast, adults aged 60–64 years (*n* = 20,599) achieved 100% appropriateness, confirming this group as the benchmark for correct administration.

Adults aged 18–59 years, both with and without chronic conditions, showed very high adherence, with ≥99% receiving appropriate vaccines and no significant inappropriate use recorded. Pregnant and postpartum women, as well as all pediatric groups, also demonstrated full adherence to the recommended formulations.

When compared with the 60–64 group, individuals aged ≥65 years were at significantly higher risk of receiving a non-recommended vaccine, with a Relative Risk (RR) of 0.23 (95% CI: 0.225–0.231; Table 2). Complete data on vaccine administrations by group and vaccine type, including both appropriate and inappropriate doses, are provided in the Supplementary Materials (Table S1).

### 3.2. 2024–2025 Campaign

**Figure 2:**
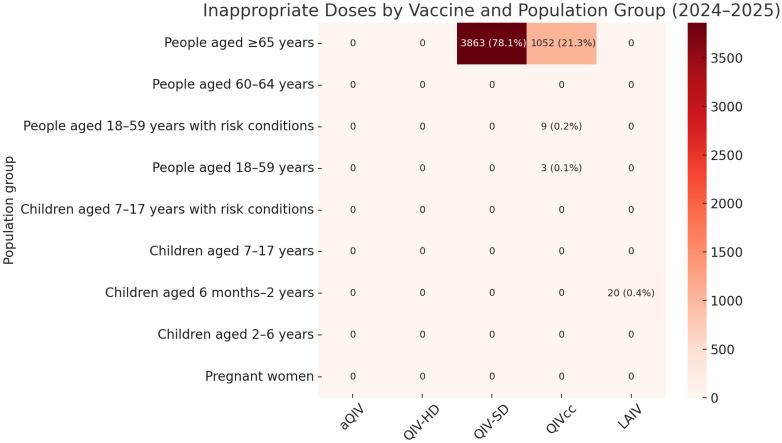
Distribution of inappropriate influenza vaccine administrations by population group and vaccine type during the 2024–2025 season. Note: each cell reports the number and percentage of non-recommended doses administered within the group. White cells indicate no inappropriate administrations.

A summary of the results for the 2024–2025 campaign is shown in Figure 2. This season demonstrated improved appropriateness across most target populations. Among individuals aged ≥65 years (*n* = 124,733), the proportion receiving inappropriate formulations decreased to 3.9% (3863 QIV-SD and 1052 QIVcc), resulting in an overall appropriateness of 96.1%. This represents a nearly fivefold reduction in the risk of inappropriate administration compared to the previous season.

Once again, adults aged 60–64 years (*n* = 18,508) achieved 100% appropriateness, serving as a stable reference category. Adults aged 18–59 years with chronic conditions (*n* = 15,098) received appropriate vaccines in 99.9% of cases, with only nine instances of LAIV being used inappropriately. Similarly, among healthy adults aged 18–59 years (*n* = 16,025), only three cases of inappropriate LAIV use were recorded.

In pediatric groups, most vaccinations adhered to recommendations. However, a critical issue was identified: among children aged 6 months–2 years (*n* = 325), 6.2% (20 children) were inappropriately administered LAIV, a formulation not authorized for this age group. Pregnant and postpartum women again showed near-perfect adherence.

Compared to the 60–64 age group, individuals aged ≥65 years in 2024–2025 were significantly less likely to receive a non-recommended formulation, with a Relative Risk (RR) of 0.12 (95% CI: 0.118–0.128; Table 2). Complete data on vaccine administrations by group and vaccine type, including both appropriate and inappropriate doses, are provided in the Supplementary Materials (Table S2).

## 4. Discussion

The findings from our study on the appropriateness of influenza vaccination conducted by the LHA of Catania during the 2023–2024 and 2024–2025 campaigns offer valuable insights for contextualizing local vaccination practices within the Italian and European context. The primary objective of current public health strategies is “precision vaccination,” which aims to align the most appropriate vaccine with an individual’s risk profile and age to maximize protection [28]. Previous works have addressed the issue of appropriateness from different perspectives. For example, Boccalini et al. conducted surveys investigating physicians’ preferences and awareness regarding age- and risk-specific vaccine formulations, highlighting the need for greater alignment between recommendations and clinical practice [29]. Other studies, such as that by Rumi et al., applied health economic and budget-impact models to estimate the benefits of stratified vaccine use in the elderly, underscoring both the clinical and economic value of targeted immunization strategies [30]. In addition, expert recommendations published by Boccalini and colleagues have emphasized the importance of precision vaccination strategies in Italy, stressing the role of tailored vaccine allocation to optimize protection in vulnerable populations [16]. Beyond these perspectives, further evidence has documented regional variability and challenges in the uptake of enhanced influenza vaccines among older adults. Pestarino et al. analyzed the rollout of the 2022/2023 vaccination campaign in Italy, reporting marked heterogeneity in the use of adjuvanted and high-dose formulations [21]. Another study highlighted spatial and temporal variability in influenza vaccination coverage among the elderly, linking differences to organizational and workload factors among general practitioners [31]. Moreover, research on missed opportunities for influenza vaccination in older adults has revealed psychological, contextual, sociodemographic and communication gaps that contribute to suboptimal uptake of recommended vaccines [32]. At the European level, expert reviews have also emphasized that while enhanced vaccines offer superior protection for older populations, their adoption remains inconsistent and often dependent on local policy and access frameworks [33]. Taken together, these contributions provide complementary perspectives that frame the rationale for our analysis of vaccine appropriateness in the local context.

### 4.1. Older Adults (≥65 Years)

Older adults represent a high-priority target group due to their elevated risk of severe complications and influenza-related mortality [18]. For this age group, national guidelines explicitly recommend aQIV or QIV-HD vaccines to enhance the immune response and counteract immunosenescence. The superior efficacy of these vaccines in reducing hospitalizations has been confirmed by systematic reviews and meta-analyses [34]. Our data for the 2023–2024 campaign in Catania, where only 78.8% of individuals aged ≥65 years (*n* = 123,281) received the recommended formulations [35], reflecting a persistent challenge also documented at both national and European levels. This finding is consistent with the framework of regional variability in the optimization of enhanced vaccine use in Italy [21] and and aligns with data showing persistently suboptimal coverage among the elderly in several EU member states [36]. The causes of this issue, as suggested by the literature, are often multifactorial and include sociodemographic factors and and varying levels of trust in the healthcare system [37]. Our calculation of a RR of 0.23 (95% CI: 0.225–0.231) for receiving a non-optimal vaccine in this group (compared to the 60–64 age group) precisely quantifies the extent of the problem in the first season analyzed. It is important to note that while the comparison with the 60–64 age group is a useful benchmark, it highlights an intrinsic challenge: the stricter recommendations for older adults (only aQIV or QIV-HD are recommended) inherently increase the likelihood of in-appropriate administrations compared to groups with broader vaccine options. The notable improvement observed in the 2024–2025 campaign, with an appropriateness of 96.1% among those over 65 (*n* = 124,733), is an extremely positive result and suggests the effectiveness of corrective strategies implemented locally. This progress aligns perfectly with the objectives of the national directive for the 2024–2025 season [38]. The drastic reduction of the RR to 0.12 (95% CI: 0.118–0.128) in the second season is an unequivocal sign of the effectiveness of the measures taken.

Beyond potential educational gaps, it is plausible that the observed differences, particularly the high rate of inappropriate administrations in the elderly during the 2023–2024 season, may have been influenced by logistical factors. Stock limitations or delays in the supply of enhanced vaccines (aQIV, QIV-HD) might have compelled providers to use standard-dose formulations to avoid vaccination delays. Although our dataset cannot distinguish the root cause, the marked reduction in such administrations in the following season suggests that, in addition to greater awareness, improved supply management may have played a role.

### 4.2. Population Aged 60–64 Years

The inclusion of adults aged 60–64 among target populations for influenza vaccination is a relatively recent policy development in Italy [36]. In both campaigns, this group in LHA of Catania achieved 100% vaccine appropriateness. This result reflects clear adherence to guidelines and a potential ease of implementation of recommendations for this group, which serves as a benchmark for the correctness of administrations. The result is in line with expectations, given that for this group the vaccine options are broader than for the elderly, making it easier to choose an appropriate vaccine from those available [39].

### 4.3. Adults (18–59 Years) with and Without Risk Conditions

Adults aged 18–59 years, both with and without risk conditions, showed very high levels of vaccine appropriateness (≥99%) in both campaigns. This is in line with current recommendations, which consider both QIV-SD and QIVcc as suitable options for this age group [38]. In the 2024–2025 season, only a few inappropriate administrations occurred, limited to 9 cases in the at-risk group and 3 in the group without risk, who received the LAIV vaccine. The near-total absence of inappropriate administration reflects effective management and understanding of the guidelines for these groups [40]. Nevertheless, the occurrence of even a few such events suggests the value of further qualitative investigations [41].

### 4.4. Pediatric Population and Pregnant/Postpartum Women

Pediatric groups and pregnant/postpartum women are key priority groups for influenza vaccination. Although immunization during pregnancy is strongly recommended to protect both mother and newborn, coverage rates in Italy have historically been below target levels, largely due to low-risk perception and the lack of a clear recommendation from healthcare providers [27,42]. In this study, vaccination among pregnant women demonstrated near-perfect adherence, suggesting strong local performance. Most pediatric subgroups also showed high appropriateness, particularly children aged 7–17 years, who predominantly received LAIV and QIV-SD in accordance with guidelines.

However, a critical issue emerged during the 2024–2025 season: 6.2% of children aged 6 months to 2 years (20 out of 325) were inappropriately administered LAIV, despite it not being authorized for this age group according to both Ministry of Health guidelines and Italian Medicines Agency (AIFA) directives [32,43]. While numerically limited, this procedural error underscores broader challenges in pediatric vaccination, such as caregiver hesitancy and the complexity of age-specific recommendations [44]. To mitigate such risks, targeted strategies—such as electronic checklists, clinical decision support systems, and ongoing provider training—should be adopted to reinforce accuracy in vaccine administration [45].

## 5. Study Limitations

This study has several limitations that should be acknowledged. First, the retrospective nature of the study relies on administrative data; consequently, its validity depends on the accuracy and completeness of data recording, and the presence of entry errors cannot be excluded. Second, our analysis focused on administrative appropriateness and did not include clinical outcomes. Therefore, we cannot draw conclusions about the direct impact of improved appropriateness on influenza-related morbidity or adverse events. Third, the dataset does not provide insight into the reasons behind inappropriate administration. Consequently, it is not possible to distinguish between logistical constraints (e.g., stockouts), clinician choice, or patient preference as the root cause. Furthermore, the dataset did not allow for stratification of the elderly population (≥65 years) into narrower age bands. Such an analysis could have revealed whether inappropriate administrations were clustered around age 65 (suggesting provider uncertainty about the age cutoff) or if they were evenly distributed. Future research should explore this granularity to more precisely identify educational or systemic gaps. In addition, our study could not stratify data by vaccination setting. This analysis would have helped identify where inappropriate administrations are concentrated and, consequently, where to target corrective interventions more effectively. This represents an important avenue for future studies. Finally, as the study was conducted within a single LHA in Catania, the findings may not be directly generalized to other regions with different healthcare systems, populations, or logistical frameworks.

## 6. Conclusions

This study highlights the capacity of an LHA to substantially improve the appropriateness of influenza vaccine administration within a relatively short time frame.

The most notable achievement was the near-elimination of suboptimal vaccine use in the elderly population (≥65 years), with rates decreasing from 21.2% in the 2023–2024 season to just 3.9% in 2024–2025. Combined with the consistently high adherence rates (≥99%) observed across nearly all other target groups, these results suggest that the corrective measures implemented were both effective and sustainable.

Nonetheless, the analysis identified a critical procedural issue: the inappropriate use of LAIV in 6.2% of children under two years of age—a group for whom this vaccine is contraindicated. This finding serves as a reminder that even high-performing systems require ongoing attention to specific risk areas to ensure comprehensive patient safety.

## Figures and Tables

**Table 1:** Recommended and available influenza vaccine types by target population.

Target Population	QIV-SD	QIVcc	aQIV	QIV-HD	LAIV
**People aged ≥65 years**	S	S	R	R	-
**People aged 60–64 years**	S	S	S	S	-
**People aged 18–59 years with risk conditions**	S	S	S	-	-
**Adults aged 18–59 years**	S	S	S	-	-
**Children aged 7–17 years with risk conditions**	S	S	-	S	S
**Children aged 7–17 years**	S	S	-	-	S
**Children aged 2–6 years**	S	S	-	-	S
**Children aged 6 months–2 years**	S	-	-	-	-
**Pregnant and postpartum women**	S	S	-	-	-

R = explicitly recommended for the specified group; S = are allowed and included in the immunization offer. - = not authorized or not suggested for that population group.

**Table 2:** RR of inappropriate influenza vaccine administration in adults ≥65 years compared to adults aged 60–64 years, 2023–2024 and 2024–2025 seasons.

Season	Population Group	*n* Vaccinated	Inappropriate Doses *n* (%)	Reference Group	RR (95% CI)
2023–2024	≥65 years	123,281	26,141 (21.2%)	Adults 60–64 years	0.23 (0.225–0.231)
	Adults 60–64 years	20,599	0 (0.0%)	—	Reference
2024–2025	≥65 years	124,733	4915 (3.9%)	Adults 60–64 years	0.12 (0.118–0.128)
	Adults 60–64 years	18,508	0 (0.0%)	—	Reference

Note: RR = Relative Risk; CI = Confidence Interval.

## Data Availability

The data of this article will be made available by the authors upon request.

## Supplementary Materials

The following are available online at https://www.mdpi.com/article/10.3390/vaccines13090925/s1, Table S1: Distribution of influenza vaccines administered by population group and vaccine type during the 2023–2024 influenza season; Table S2: Distribution of influenza vaccines administered by population group and vaccine type during the 2024–2025 influenza season.

## Abbreviations

aQIV = adjuvanted quadrivalent inactivated vaccine; ARDS = acute respiratory distress syndrome; LAIV = live attenuated intranasal vaccine; LHA = Local Health Authority; QIVcc = cell-based quadrivalent inactivated vaccine; QIV-HD = high-dose quadrivalent inactivated vaccine; QIV-SD = standard-dose quadrivalent inactivated vaccine.
