# Supplementary material for: Influenza Vaccination Appropriateness: Insights from the Local Health Unit of Catania During the 2023/2024 and 2024/2025 Seasons
Source: Vaccines (Basel). 2025 Aug 30;13(9):925. doi: 10.3390/vaccines13090925 (PMC12474316; doi:10.3390/vaccines13090925)
Supplement: Supplementary file 1 [file vaccines-13-00925-s001.zip › vaccines-3775817-supplementary.pdf]

Table S1. Distribution of influenza vaccines administered by population group and vaccine type during the 2023–2024 influenza season. Inappropriate administrations marked with *.

Population group	aQIV, n (%)		QIV-HD, n (%)		QIV-SD, n (%)		QIVcc, n (%)		LAIV, n (%)		Total,	Median (±SD)	
People aged ≥65 years	72744	(59,0)	24396	(19,8)	**25987***	**(21,1)**	**154***	**(0,1)**	0	(0,0)	**123281**	25191,5	(±30346,1)
People aged 60–64 years	0	(0,0)	1444	(7,0)	19001	(92,2)	154	(0,7)	0	(0,0)	**20599**	1444	(±9256,8)
People aged 18–59 years with risk conditions	0	(0,0)	0	(0,0)	23063	(95,3)	1140	(4,7)	0	(0,0)	**24203**	12101,5	(±12998,8)
People aged 18–59 years	0	(0,0)	0	(0,0)	22920	(99,2)	181	0,80	0	(0,0)	**23101**	11550,5	(±13180,9)
Children aged 7–17 years with risk conditions	0	(0,0)	0	(00)	860	(65,4)	0	(0,0)	454	(34,6)	**1314**	657	(±430,2)
Children aged 7–17 years	0	(0,0)	0	(0,0)	994	(47,4)	0	(0,0)	1104	(52,6)	**2098**	1049	(±608,1)
Children aged 6 months–2 years	0	(0,0)	0	(0,0)	441	(100,0)	0	(0,0)	0	(0,0)	**441**	441	(±254,6)
Children aged 2–6 years	0	(0,0)	0	(0,0)	881	(33,5)	0	(0,0)	1751	(66,5)	**2632**	1316	(±875,5)
Pregnant women	0	(0,0)	0	(0,0)	247	(84,0)	47	(16,0)	0	(0,0)	**294**	147	(±141,4)
Total	**72744**	**(36,7)**	**25840**	**(13,1)**	**94394**	**(47,7)**	**1676**	**(0,8)**	**3309**	**(1,7)**	**197963**	25840	(±41968,8)

Table S2. Distribution of influenza vaccines administered by population group and vaccine type during the 2024–2025 influenza season. Inappropriate administrations marked with *.

**Population group**	**aQIV, n (%)**		**QIV_HD, n (%)**		**QIV-SD, n (%)**		**QIVcc, n (%)**		**LAIV, n (%)**		**Total, n**	**Median (±SD)**	
People aged ≥65 years	69234	(55,5)	50584	(40,6)	**3863***	**(3,1)**	**1052***	**(0,8)**	0	(0,0)	**124733**	27223,5	(±34051,6)
People aged 60–64 years	8931	(48,3)	3586	(19,4)	5189	(28,0)	802	(4,3)	0	(0,0)	**18508**	4387,5	(±3393,8)
People aged 18–59 years with risk conditions	0	(0,0)	0	(0,0)	11946	(79,1)	3143	(20,8)	**9***	**(0,1)**	**15098**	7544,5	(±6188,8)
People aged 18–59 years	0	(0,0)	0	(0,0)	13153	(82,1)	2869	(17,9)	**3***	**(0,0)**	**16025**	2869	(±6914,9)
Children aged 7–17 years with risk conditions	0	(0,0)	0	(0,0)	531	(50,5)	94	(8,9)	427	(40,6)	**1052**	427	(±228,3)
Children aged 7–17 years	0	(0,0)	0	(0,0)	698	(36,4)	98	(5,1)	1122	(58,5)	**1918**	698	(±514,5)
Children aged 6 months–2 years	0	(0,0)	0	(0,0)	305	(93,8)	0	(0,0)	**20***	**(6,2)**	**325**	162,5	(±170,6)
Children aged 2–6 years	0	(0,0)	0	(0,0)	693	(22,1)	17	(0,5)	2428	(77,4)	**3138**	693	(±1243,7)
Pregnant women	0	(0,0)	0	(0,0)	146	(49,2)	151	(50,8)	0	(0,0)	**297**	148,5	(±3,5)
Total	**78165**	**(43,2)**	**54170**	**(29,9)**	**36524**	**(20,2)**	**8226**	**(4,5)**	**4009**	**(2,2)**	**181094**	36524	(±31236,6)
